# Paediatric Otorhinolaryngological Disorders: Early Detection and Management Strategies

**DOI:** 10.7759/cureus.97004

**Published:** 2025-11-16

**Authors:** Sayyed Muddasir Shah, Mubashar Ullah Jan, Majid Ali, Haider Zaman, Bilal Khan, Rahman Shar

**Affiliations:** 1 Department of ENT, Mardan Medical Complex, Bacha Khan Medical College, Mardan, PAK

**Keywords:** adenoid hypertrophy, early detection, otitis media, outpatient care, pediatric ent, tonsillitis

## Abstract

Background: Pediatric ear, nose, and throat (ENT) disorders are common and can affect hearing, speech development, sleep quality, and overall well-being. Early identification and appropriate management are essential to prevent complications and promote healthy development.

Objective: To determine the prevalence of common pediatric ENT disorders presenting to an outpatient clinic and to describe the diagnostic and management strategies used in this local clinical setting.

Methodology: This prospective observational study was conducted over 12 months in the outpatient Department of ENT at Mardan Medical Complex, Bacha Khan Medical College (MMC/BKMC), Mardan. A total of 100 children aged 0-14 years with clinically confirmed pediatric otorhinolaryngological disorders were included. Diagnosis was based on clinical examination, otoscopic and nasal assessment, and audiometry or imaging when indicated. Management consisted of medical treatment, surgical intervention where required, and scheduled follow-up visits to monitor short-term outcomes.

Results: The most frequent conditions observed were otitis media (30, 30%), tonsillitis (25, 25%), adenoid hypertrophy (20, 20%), allergic rhinitis (15, 15%), pediatric rhinosinusitis (5, 5%), and *upper aerodigestive *foreign bodies in the ear or nasal cavity/upper aerodigestive tract (5, 5%). Medical treatment effectively resolved symptoms in most infection-related cases, while surgical procedures such as adenoidectomy, tonsillectomy, and tympanostomy tube insertion improved airway obstruction and reduced recurrence. Supportive measures, including speech and nutritional counseling, contributed to developmental improvement in selected cases.

Conclusions: Common pediatric ENT disorders can be effectively managed with prompt clinical evaluation and appropriate intervention. Increasing caregiver awareness and ensuring consistent access to ENT diagnostic and treatment services at the local healthcare level can help prevent avoidable complications and support better long-term outcomes.

## Introduction

Pediatric otorhinolaryngological (ENT) disorders are among the most common reasons for outpatient and emergency consultations in children and may significantly affect hearing, speech development, sleep quality, school performance, and overall growth [1-2]. The most frequently encountered pediatric ENT conditions include otitis media, recurrent tonsillitis, adenoid hypertrophy, allergic rhinitis, and chronic sinusitis [3]. Early recognition and timely management are essential to prevent long-term complications during critical developmental periods [4].

Recurrent otitis media, if untreated, can lead to conductive hearing loss, adversely affecting speech and language acquisition [5]. Similarly, adenoid hypertrophy and enlarged tonsils contribute to sleep-disordered breathing, behavioral issues, impaired school performance, and growth delay [6]. Allergic rhinitis may predispose to chronic nasal obstruction and recurrent sinus infections, affecting daily functioning and quality of life [7].

Evaluation of pediatric ENT disorders generally includes otoscopic examination, audiological testing, tympanometry, and nasopharyngeal endoscopy, with imaging reserved for selected presentations [8]. Although these diagnostic tools are well-established, their consistent availability may vary across different regional healthcare settings, particularly in resource-limited systems [9].

Management strategies range from medical therapy (antibiotics, nasal corticosteroids, antihistamines, analgesics) to surgical interventions such as adenoidectomy, tonsillectomy, and myringotomy with ventilation tube insertion when clinically indicated [10]. Removal of foreign bodies from the ear, nose, or upper aerodigestive tract is also a common pediatric ENT procedure [11]. Supportive measures such as speech therapy and nutritional monitoring may further aid recovery and developmental progress [12-13].

Given the clinical burden of these disorders and the importance of early intervention, evaluating their prevalence and management patterns within local healthcare settings is important. The objective of this study is to determine the prevalence and clinical characteristics of common pediatric ENT disorders presenting to Mardan Medical Complex, Bacha Khan Medical College (MMC, BKMC), Mardan, and describe the diagnostic and management approaches used.

Research objectives

To determine the prevalence of pediatric ENT disorders, it is necessary to analyze the effectiveness of early detection and outcomes of medical and surgical treatments on the prognosis and quality of life of children.

## Materials and methods

Study design and setting 

This prospective descriptive study was conducted in the Department of Otolaryngology-Head and Neck Surgery, MTI Mardan Medical Complex (MMC), Mardan, Khyber Pakhtunkhwa, Pakistan, over a period of 12 months (January 2025 to January 2026). A total of 100 pediatric patients (aged ≤14 years) presenting with clinically diagnosed ENT disorders were included. Both outpatient (OPD) visits and acute presentations from the Emergency Department were enrolled, provided the diagnosis was confirmed by an ENT specialist. Patients who required further inpatient management were admitted and followed accordingly.

Participants

A total of 100 pediatric patients (mean age 0-14 years) with clinically confirmed ENT disorders were included in this study. Children with otitis media, tonsillitis, adenoid hypertrophy, allergic rhinitis, sinusitis, and foreign body ingestion qualified for the study. Patients with systemic comorbidities and incomplete data were excluded from the analysis. 

Sample size calculation

The minimum required sample size was calculated using the formula *n* = *Z*² × *P*(1 - *P*)/*d*² [14], assuming an estimated prevalence (*P*) of 30% for pediatric ENT disorders from prior regional studies, a 95% confidence level (*Z* = 1.96), and a 9% margin of error (*d* = 0.09). The calculated sample size was 89; thus, 100 patients were included to improve study power.

Inclusion criteria

Children with systemic illnesses affecting the ear, nose, or throat (e.g., syndromic craniofacial anomalies), lower respiratory tract diseases such as croup, congenital airway abnormalities such as laryngomalacia, and conditions requiring specialized pediatric airway management were excluded, as these are primarily managed under pediatric airway or pediatric surgical care rather than general ENT outpatient services.

Exclusion criteria

Participants presenting with systemic comorbidities, incomplete medical records, or a history of prior otolaryngological surgeries were systematically excluded from the study.

Diagnostic and management strategy

Clinical information was obtained through detailed history-taking from caregivers, physical examination, otoscopic evaluation, and nasopharyngeal assessment. Audiometric testing and tympanometry were performed where indicated, and imaging studies were utilized selectively (e.g., suspected sinusitis or adenoid hypertrophy).Management strategies were planned according to established pediatric ENT guidelines. Medical therapy included age-appropriate antibiotics, analgesics, antihistamines, and intranasal corticosteroids where clinically indicated. Nasal saline irrigation was recommended for children of all ages. Topical nasal decongestants were used cautiously and only in children aged over 6 years, and for short durations (maximum 3-5 days) to avoid rebound congestion. Surgical procedures, including adenoidectomy, tonsillectomy, and myringotomy with tympanostomy tube insertion, were reserved for cases not responding to medical therapy or those presenting with obstructive or recurrent disease patterns. Supportive care, including speech therapy and nutritional counseling, was provided when needed. Patients were monitored through scheduled follow-up visits to evaluate symptom resolution and developmental progress.

Statistical analysis

The data were processed and analyzed with IBM SPSS Statistics for Windows, Version 20.0 (IBM Corp., Armonk, NY) [15]. Descriptive data were presented as mean ± SD, frequency, and percentage. The association between the outcomes and the relevant interventions was computed using the chi-square test. A *P*-value < 0.05 was considered statistically significant. 

**Figure 1:**
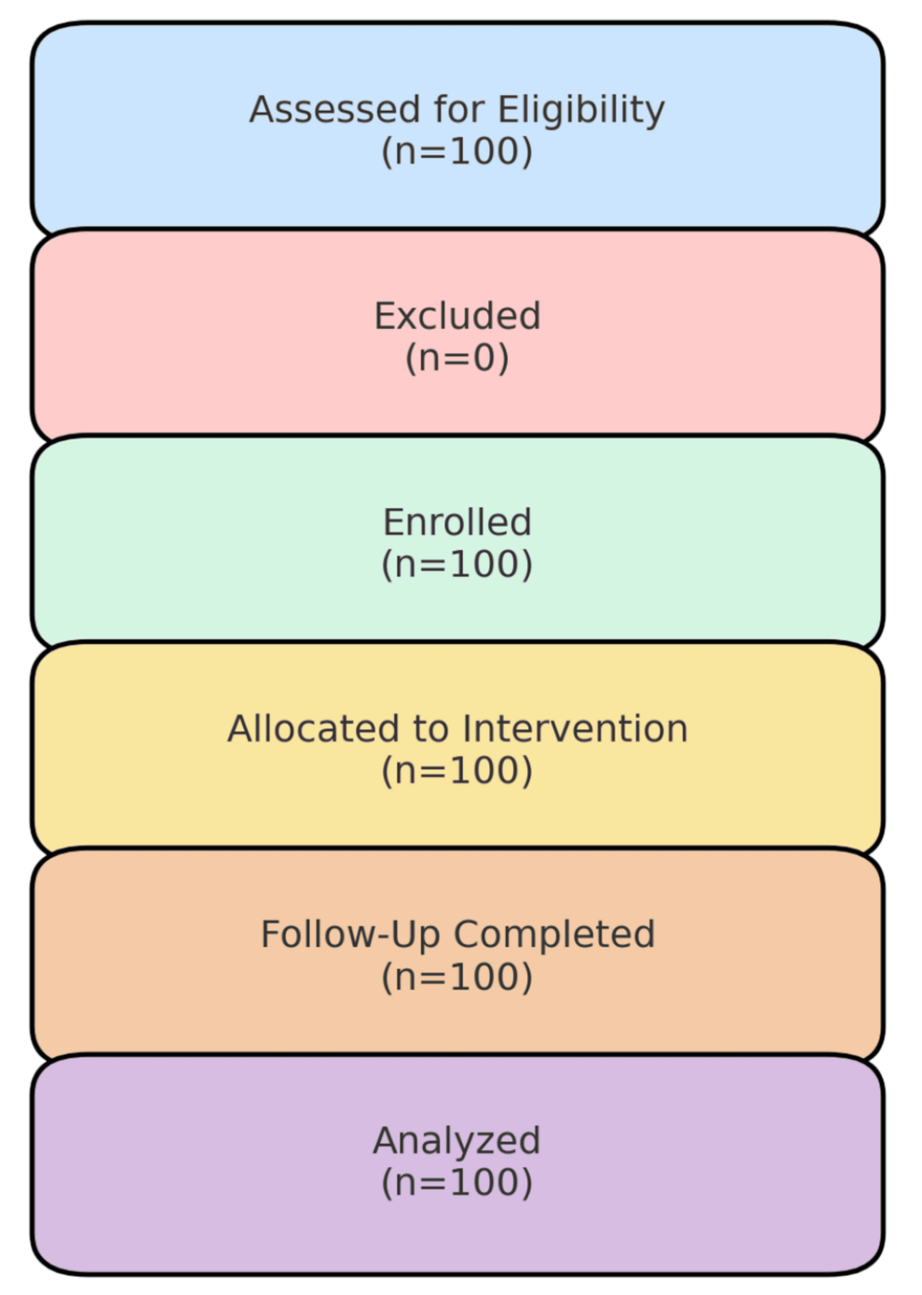
Participant flow diagram. This figure illustrates the flow of pediatric patients through the study. A total of 100 consecutive eligible children presenting to the ENT outpatient and emergency services during the study period were assessed. Patients were enrolled only after meeting the inclusion criteria and confirming an ENT diagnosis. No patients who met the inclusion criteria declined participation or were lost to follow-up; therefore, all 100 children were included in the final analysis. Image credit: All authors.

Figure 1 shows the flow of pediatric participants in the study. One hundred eligible children were registered, and all completed the follow-up period. Thus, all children were part of the final analysis.

Table 1 shows the baseline demographics of the study population distributed by the study ages, and most patients were between three and eight years of age.

**Table 1:** Baseline demographics of pediatric patients (N = 100). Mean age = 7.4 ± 3.5 years.

Age group (years)	n	%
0-2	20	20
3-5	25	25
6-8	20	20
9-11	15	15
12-14	20	20

Table 2 describes the prevalence of the key ENT disorders affecting children. Otitis media was portrayed most prevalently, followed by tonsillitis and adenoid hypertrophy.

**Table 2:** Prevalence of ENT disorders among pediatric patients (N = 100). Only foreign bodies located in the ear, nasal cavity, or upper aerodigestive tract were included in this study. Gastrointestinal foreign body ingestion and lower airway foreign body aspiration were excluded, as these fall outside routine ENT management.

ENT disorder	n	%
Otitis media	30	30%
Recurrent tonsillitis	25	25%
Adenoid hypertrophy	20	20%
Allergic rhinitis	15	15%
Chronic sinusitis	5	5%
Foreign body in ear, nose, or upper aerodigestive tract	5	5%

Table 3 outlines the management strategies applied, including medical, surgical, and supportive interventions, along with the corresponding clinical improvement observed during follow-up. The outcomes demonstrate that the majority of patients showed measurable symptomatic improvement following appropriate treatment. The summarized findings are presented in Figure 1 and Tables 1-3. Data were collected using a structured parent questionnaire and clinician form (Appendix).

**Table 3:** Management strategies and clinical outcomes in pediatric ENT disorders (N = 100). Improvement rates are expressed as *n* (%).

Management strategy	Patients (*n*)	Percentage (%)	Improvement rate, *n* (%)	Statistical test (*P*-value)
Antibiotics for infections (e.g., otitis media, sinusitis)	45	45	27 (90%)	χ² = 12.5, *P* = 0.001
Antihistamines and nasal sprays (allergic rhinitis)	15	15	11 (75%)	χ² = 8.2, *P* = 0.004
Surgical interventions (tonsillectomy, adenoidectomy, myringotomy with tube insertion)	35	35	30 (85%)	χ² = 15.3, *P* < 0.001
Supportive care (speech therapy, nutritional support)	5	5	4 (80%)	Fisher’s exact test, *P* = 0.021
Total	100	100	-	-

Ethical approval statement 

Ethical approval was obtained from the Institutional Review Board (IRB) of BKMC/MMC, Mardan (IRB No. 471/DOS/BKMC/MMC). Written informed consent was obtained from parents or legal guardians of all participants.

## Results

A total of 100 pediatric patients were included (mean age 7.1 ± 3.6 years); the gender distribution was 56 (56%) male and 44 (44%) female.

Prevalence of disorders

Otitis media (30, 30%) was the most common disorder, followed by tonsillitis (25, 25%), adenoid hypertrophy (20, 20%), allergic rhinitis (15, 15%), sinusitis (5, 5%), and foreign body ingestion (5, 5%). These medical conditions were identified at an early stage through systematic clinical evaluations and parental guidance.

Intervention outcomes

Antibiotic therapy led to clinical resolution of symptoms in 27 out of 30 children with otitis media (90%), as evidenced by the resolution of otalgia and fever, along with normalization of otoscopic findings during follow-up. Adenoidectomy resulted in noticeable improvement in nasal obstruction and nighttime breathing in 17 out of 20 patients (85%), based on clinical examination and caregiver-reported sleep and breathing patterns; no formal polysomnography was performed. Antihistamines and intranasal corticosteroids alleviated nasal congestion and sneezing in 11 out of 15 patients with allergic rhinitis (75%). Surgical interventions, including tonsillectomy and myringotomy with tympanostomy tube placement, were associated with a reduction in recurrence frequency and improved symptom control. Supportive interventions, such as speech therapy and nutritional counseling, contributed to developmental progress in 4 out of 5 children receiving these services (80%). Most patients maintained stable growth and sustained clinical improvement on follow-up.

## Discussion

This study found that otitis media, recurrent tonsillitis, adenoid hypertrophy, allergic rhinitis, and chronic sinusitis were the most common pediatric ENT conditions presenting in our clinical setting. This pattern is consistent with previously reported distributions of pediatric ENT disorders in regional and international studies. These conditions are known to affect hearing, sleep quality, feeding, and overall development when not identified and managed appropriately [16].

Antibiotic therapy for otitis media and sinusitis resulted in symptomatic improvement in most of the affected children, in line with current pediatric practice recommendations [17]. Surgical management, including adenoidectomy, tonsillectomy, and myringotomy with tympanostomy tube insertion, was offered when recurrent infections or obstructive symptoms persisted despite medical treatment. These interventions are well-established treatment options for selected cases and have been shown to reduce disease burden and improve functional outcomes [18].

Foreign bodies in the ear, nose, and upper aerodigestive tract represented a smaller but clinically important proportion of cases. Their prompt removal is necessary to prevent airway compromise, infection, and mucosal injury, and is a routine part of pediatric ENT practice [19].

Follow-up in this study was limited to six months, during which most children demonstrated clinical improvement based on symptom resolution and caregiver reports. However, many pediatric ENT conditions-particularly recurrent otitis media, allergic rhinitis, and adenotonsillar disease-may follow a prolonged or fluctuating course. Therefore, long-term outcomes, recurrence patterns, hearing assessments, and developmental effects could not be evaluated within the timeframe of this study. Longer follow-up periods are needed to determine the persistence of improvement over time.

Access to timely ENT evaluation varies across healthcare settings. Strengthening local-level pathways for early diagnosis, parental education, and referral systems may reduce delayed presentation and prevent chronic complications [20-21].

Limitations

This study was conducted at a single tertiary-care center with a moderate sample size, which may limit generalizability. The follow-up period was limited to six months, and therefore, long-term recurrence, hearing outcomes, and developmental progression could not be assessed. Variations in local diagnostic resources may have influenced case recognition and intervention timing.

## Conclusions

Common pediatric ENT disorders can be effectively managed when recognized early and treated appropriately. Improving local caregiver awareness and consistency of access to ENT services may help reduce preventable complications. Future studies with larger cohorts and long-term follow-up are required to better understand recurrence patterns and developmental outcomes.

## Appendices

Appendix 

**Table 4:** Data Collection and Parental Questionnaire Form Appendix Table 1 shows the integrated parent questionnaire and clinician data collection form utilized in the study. It encompasses demographics, medical history, awareness and care-seeking behaviors, clinical manifestations, diagnostic confirmation, investigations, management plans, and follow-up outcomes. This type of supplementary material is indispensable for verifying the systematic and reproducible nature of the study findings.

Section	Item	Options / Response
Demographics	Child’s Age	______ years
	Sex	☐ Male ☐ Female
	Residence	☐ Urban ☐ Rural
	Parental Education	☐ No formal ☐ Primary ☐ Secondary ☐ Higher
Medical History	Previous ENT Diagnosis	☐ Otitis Media ☐ Tonsillitis ☐ Adenoid Hypertrophy ☐ Allergic Rhinitis ☐ Sinusitis ☐ Foreign Body Ingestion
	Recurrent Infections in Last 12 Months	☐ Yes ☐ No
Awareness & Care-Seeking	Awareness of Routine ENT Screening	☐ Yes ☐ No
	Sought Medical Help Promptly	☐ Yes ☐ No
	Barriers to Care	☐ Lack of awareness ☐ Financial cost ☐ Accessibility ☐ Fear of procedures
Clinical Features (Exam)	Presenting Symptoms	☐ Fever ☐ Chills ☐ Ear Discharge ☐ Nasal Obstruction ☐ Snoring/Sleep Apnea ☐ Headache ☐ Hearing Loss ☐ Other: ____
Diagnosis	Confirmed ENT Disorder	☐ Otitis Media ☐ Tonsillitis ☐ Adenoid Hypertrophy ☐ Allergic Rhinitis ☐ Sinusitis ☐ Foreign Body Ingestion
Investigations	Tests Performed	☐ Audiogram ☐ Imaging (X-ray/CT/MRI) ☐ Other: ____
Management Plan	Treatment Given	☐ Antibiotics ☐ Antihistamines/Nasal Sprays ☐ Surgical Intervention ☐ Supportive Care
Follow-up	Outcome	☐ Improved ☐ No Change ☐ Worsened
